# Goals and Challenges in Bacterial Phosphoproteomics

**DOI:** 10.3390/ijms20225678

**Published:** 2019-11-13

**Authors:** Paula Yagüe, Nathaly Gonzalez-Quiñonez, Gemma Fernánez-García, Sergio Alonso-Fernández, Angel Manteca

**Affiliations:** Área de Microbiología, Departamento de Biología Funcional, IUOPA, ISPA, Facultad de Medicina, Universidad de Oviedo, 33006 Oviedo, Spain; paula.yague@gmail.com (P.Y.); natygq@gmail.com (N.G.-Q.); gemmafg06@hotmail.com (G.F.-G.); sergioalonsofernandez@gmail.com (S.A.-F.)

**Keywords:** bacteria, Ser/Thr/Tyr phosphorylation, His phosphorylation, phosphoproteomics, differentiation, sporulation, LC-MS/MS

## Abstract

Reversible protein phosphorylation at serine, threonine and tyrosine is a well-known dynamic post-translational modification with stunning regulatory and signalling functions in eukaryotes. Shotgun phosphoproteomic analyses revealed that this post-translational modification is dramatically lower in bacteria than in eukaryotes. However, Ser/Thr/Tyr phosphorylation is present in all analysed bacteria (24 eubacteria and 1 archaea). It affects central processes, such as primary and secondary metabolism development, sporulation, pathogenicity, virulence or antibiotic resistance. Twenty-nine phosphoprotein orthologues were systematically identified in bacteria: ribosomal proteins, enzymes from glycolysis and gluconeogenesis, elongation factors, cell division proteins, RNA polymerases, ATP synthases and enzymes from the citrate cycle. While Ser/Thr/Tyr phosphorylation exists in bacteria, there is a consensus that histidine phosphorylation is the most abundant protein phosphorylation in prokaryotes. Unfortunately, histidine shotgun phosphorproteomics is not possible due to the reduced phosphohistidine half-life under the acidic pH conditions used in standard LC-MS/MS analysis. However, considering the fast and continuous advances in LC-MS/MS-based phosphoproteomic methodologies, it is expected that further innovations will allow for the study of His phosphoproteomes and a better coverage of bacterial phosphoproteomes. The characterisation of the biological role of bacterial Ser/Thr/Tyr and His phosphorylations might revolutionise our understanding of prokaryotic physiology.

## 1. Introduction

Phosphoproteomics involves the analysis of a complete set of phosphorylation sites present in a cell. It has undergone a revolution since 2000, thanks to the advances in mass spectrometry (MS) based phosphoproteome methodologies. Large datasets describing the phosphoproteomes of several organisms were created. While nine amino acids (Ser, Thr, Tyr, His, Lys, Arg, Asp, Glu and Cys) can be modified by four types of phosphate protein linkages, only the phosphorylations at Ser, Thr and Tyr have been extensively characterised and associated with stunning regulatory and signalling cellular functions, especially in eukaryotes [1]. For instance, the human phosphoproteome harbours more than 30,000 Ser/Thr/Tyr phosphorylation sites [2]. Bacterial proteins can also be phosphorylated at Ser/Thr/Tyr, but to a much lesser extent. To date, 38 Ser/Thr/Tyr phosphoprotemic studies on bacteria have been reported, describing the phosphoproteome of 24 species of eubacteria and one species of archaea (*Halobacterium salinarum*) (Table 1). *Mycobacterium tuberculosis* harbours the largest bacterial pohosphoproteome described as consisting of 500 Ser/Thr/Tyr phosphorylation sites from 257 proteins [3]. Bacterial Ser and Thr phosphorylation (average abundances of 59% and 34.1% for Ser and Thr, respectively) is much more abundant than Tyr phosphorylation (average abundance of 9.9%) (Table 1). While Ser/Thr/Tyr phosphorylation exists in bacteria, there is a consensus about that histidine phosphorylation is the most abundant protein phosphorylation in prokaryotes [4]. However, these residues have almost not been subjected to phosphoproteomic analyses. There have only been three studies describing bacterial histidine phosphoproteomes [5,6,7]. This is a consequence of the acid lability of the histidine phosphate linkage, which is not compatible with most of the proteomic liquid chromatography tandem–mass spectrometry (LC-MS/MS) protocols. Protein phosphorylation at amino acid residues, other than Ser/Thr/Tyr or His, is less abundant and has been poorly characterised. In this work, we review the state of the art and the challenges of bacterial Ser/Thr/Tyr and His phosphoproteomics.

## 2. Bacterial Ser/Thr/Tyr Phosphoproteomics

Classical protein chemistry phosphoproteomic approaches require protein purification, peptide mapping and identification of phosphorylated peptide regions and sites by N-terminal sequence analysis. These kinds of analyses are tedious, and they could cover only one phosphoprotein or a few phosphoproteins. Chemistry approaches basically disappeared with the development of 2DE gel-based analyses, combined with MS or MS/MS analysis for protein identification. The most recent advances in LC-MS/MS make these traditional chemistry approaches obsolete. In this review, we describe the state of the art of a large bacterial dataset of Ser/Thr/Tyr phosphorylation identified using gel-based and LC-MS/MS-based methodologies.

### 2.1. Gel-Based Analyses

Classically, proteomics and phosphoproteomics are based on the use of 2DE gels. 2DE gel-based phosphoproteomic experiments use specific dyes [43] or antibodies [44] to identify and quantify phosphorylated protein spots. These methodologies are still useful, particularly in identifying possible isoforms of phosphorylated proteins [45]. However, due to the reduced bacterial Ser/Thr/Tyr phosphorylation, only a few reports describing bacterial Ser/Thr/Tyr phosphoproteomes by means of 2DE gel approaches have been reported. The phosphoproteomes of *Neisseria meningitidis*, *Staphylococcus aureus* and *Chlamydia caviae* were characterised by means of 2DE gel approaches [16,26,32] (Table 2).

### 2.2. LC-MS/MS-Based Phosphoproteomic Analyses

Most phosphopeptide enrichment protocols use immobilised metal affinity chromatography (IMAC), which consists positively charged metal ions, such as Fe (3+), Ga (3+), Al (3+), Zr (4+) and Ti(4+) [46]. The most widespread method is the use of TiO_2_ affinity chromatography [46]. TiO_2_ affinity chromatography-based phosphoproteomics is mainly optimised for eukaryotic samples. Further work on optimising this method to study the relatively low Ser/Thr/Tyr phosphorylation present in bacteria will contribute to deepen the characterisation of bacterial phosphoproteomes. In this sense, an interesting phosphopeptide pre-enrichment method, which largely enhances TiO_2_ efficiency, is the use of calcium phosphate precipitation (CPP) [47]. CPP consists of coprecipitated phosphorylated tryptic peptides with calcium phosphate at high pH levels [47]. CPP-pre-enriched samples are used for IMAC, enhancing the amount of purified phosphopeptides, which are further identified by LC-MS/MS analysis [47]. CPP has been successfully used in several eukaryotes, including humans [48,49], mice [50], plants [47] and yeasts [51]. CPP phosphopeptide pre-enrichment is also used in bacterial phosphoproteomics [17,40]. In *Streptomyces coelicolor*, CPP pre-enrichment increases TiO_2_ LC-MS/MS-based phosphopeptide identification by five times [17].

#### 2.2.1. Bacterial Ser/Thr/Tyr Nonquantitative LC-MS/MS-Based Phosphoproteomic Analyses

Due to low levels of Ser/Thr/Tyr bacterial phosphorylation, most bacterial Ser/Thr/Tyr phosphoproteomic studies used large amounts of protein (milligrams), obtained during the vegetative growth phase, to detect a relatively low number of phosphopeptides [17]. The aim of these studies was to identify as many phosphosites as possible, and they do not provide information about the dynamic of this phosphorylation during bacterial development. Twenty-four phosphoproteomes from 18 eubacterial species and an Archaeon (*Halobacterium salinarum*) were analysed using nonquantitative LC-MS/MS-based phosphoproteomic approaches (Table 3). These studies, together with the 2DE gel-based studies described above, were pioneering in the characterisation of the existence of Ser/Thr/Tyr phosphorylation in bacteria, rather than in the characterisation of the variation of phosphoproteomes during their development or in response to different stimuli.

#### 2.2.2. Bacterial Ser/Thr/Tyr LC-MS/MS-Based Quantitative Phosphoproteomic Analyses

Once the existence of bacterial Ser/Thr/Tyr phosphorylation was demonstrated, the next issue to be explored was whether the bacterial phosphorylation changed during bacterial differentiation and/or in response to different developmental conditions. As stated above, phosphorylation in bacteria is dramatically lower than that in eukaryotes, making bacterial phosphoproteomics challenging, especially quantitative phosphoproteomics (i.e., analyses of the amount of specific phosphorylation sites and how they vary during development). To our knowledge, there are 15 reported quantitative phosphoproteomic studies on bacteria [6,15,17,25,29,31,34,35,36,37,38,40,41,42] (Table 4).

The first bacterial quantitative phosphoproteomic study was performed in 2010 on *Bacillus subtilis* (*B. subtilis*) using the stable isotope labelling of amino acids (SILAC) in a cell culture, describing the changes in the *B. subtilis* phosphoproteome in different media [15]. In 2014, another SILAC analysis was also performed on *B. subtilis*, analysing different developmental stages [31]. Other quantitative phosphoproteomic analyses using SILAC were performed on *Escherichia coli* (*E. coli*) and *Listeria monocytogenes* [25,29].

In 2011, we performed the first quantitative phosphoproteomic study describing the differences in a bacterium during development [17]. We used CPP combined with TiO_2_ chromatography and LC-MS/MS to analyse *Streptomyces coelicolor* (*S. coelicolor*) Ser/Thr/Tyr phosphorylation [17]. This methodology was successful in identifying a relatively large amount of phosphorylation (127 phosphoproteins and 289 phosphorylation sites) from a relatively low protein amount (0.3 mg) [17]. Later, in 2018, our group improved the *S. coelicolor* quantitative phosphoproteome analysis by applying tandem mass tag (TMT) isobaric labelling to the protein extracts, prior to CPP/TiO_2_ phosphopeptide enrichment and LC-MS/MS analysis [40]. Protein and phosphoprotein abundance quantification was highly improved [40]. However, phosphopeptide identification was reduced to 48 phosphoproteins [40], while 127 phosphoproteins were identified in our previous label-free analysis [17]. The lower efficiency in phosphopeptide identification in the TMT analysis [17] was probably a consequence of mixing the very low phosphorylated vegetative samples with the more highly phosphorylated reproductive stages. TMT isobaric labelling was later used to quantify the phosphoproteome variation in virulent and nonvirulent *Mycobacterium tuberculosis* strains [38]. Dimethyl labelling was also used to analyse the variation of bacterial phosphoproteomes in *Synechocystis* sp. and *Streptococcus thermophilus* [36,42].

Label-free quantitative phosphoproteomic analyses were also performed in *Bacillus subtilis*, *Acinetobacter baumannii*, *Mycobacterium smegmatis* and *Zymomonas mobilis* [6,35,37,41]. Scheduled multiple reaction monitoring (sMRM), another label-free approach that consists in selecting the masses of the ions to be sequenced in the MS/MS, was used to analyse the *E. coli* and *Saccharopolyspora erythraea* phosphoprotoemes [30,34].

## 3. Bacterial Proteins and Pathways Modulated by Ser/Thr/Tyr Phosphorylation

### 3.1. Bacterial Proteins Identified as Phosphorylated

Bacterial cellular processes including proteins identified as phosphorylated comprise carbon/protein/nucleotide metabolism, transcription, translation, protein/cell envelope biosynthesis, two-component signalling pathways, stress response, transport or extracellular proteins (Table 2, Table 3 and Table 4). These results suggest a role of Ser/Thr/Tyr phosphorylation in the regulation of central metabolism. Proteins participating in nonessential but clinically and industrially relevant cellular activities were also identified as phosphorylated. The phosphoproteomes of the pathogenic bacteria, *Staphylococcus aureus* [26], *Chlamidia caviae* [32], *Klebsiella pneumoniae* [7], *Streptococcus pneumoniae* [14], *Helicobacter pylori* [19], *Acinetobacter baumanii* [6,27] and *Mycobacterium tuberculosis* [38], include proteins related to pathogenicity and virulence as they are capsule biosynthetic proteins, proteins involved in drug resistance or proteins related to motility. *Streptomyces*, the most important source for bioactive secondary metabolites in nature (mainly antibiotics, but also antitumorals, immunosupressors, etc.) [52], harbours Ser/Thr/Tyr-phosphorylated proteins that are involved in secondary metabolism regulation, suggesting a role of Ser/Thr/Tyr-modulating antibiotic production [17,40].

**Figure 1:**
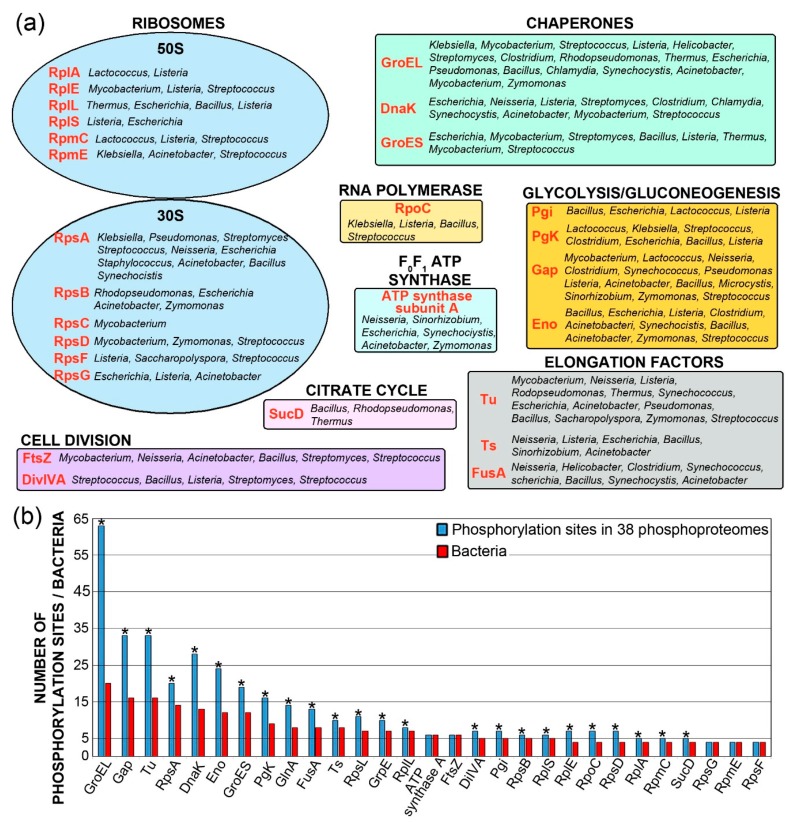
The phosphoprotein orthologues systematically identified in the 38 bacterial phosphoproteomic studies are shown in Table 1, Table 2, Table 3 and Table 4. (**a**) Phosphoproteins (highlighted in red) are classified by function. The bacteria, in which they were identified, are indicated. (**b**) Total number of the phosphorylation sites identified for each phosphoprotein orthologue in the 38 analysed bacteria, as well as the number of bacteria, in which a phosphoprotein was detected. Asterisks indicate multiphosphorylated proteins, i.e., these showing more phosphorylation sites than bacteria.

Bacteria are the most diverse group of living beings on the planet. Consequently, finding and comparing protein orthologues is not always possible. However, when we compared the 38 bacterial phosphoproteomes already known (Table 2, Table 3 and Table 4), we were able to identify 29 phosphoprotein orthologues present in at least four phosphoproteomes. These 29 phosphoproteins include 12 ribosomal proteins, four enzymes from glycolysis and gluconeogenesis, three elongation factors, two cell division proteins, one RNA polymerase subunit, one ATP synthase subunit and one enzyme from the citrate cycle (Figure 1a). Consequently, Ser/Thr/Tyr phosphorylation might modulate transcription, translation, stress response, central metabolism (glycolysis, gluconeogenesis and citrate cycle), energy production (oxidative phosphorylation) and cell division. Interestingly, the most commonly identified phosphorylated bacterial protein is the GroEL chaperone, showing 63 phosphorylation sites in 20 phosphoproteomes (Figure 1b) from 17 bacterial species. This corresponds to an average of three phosphorylation sites per protein.

### 3.2. Bacterial Ser/Thr/Tyr Phosphorylation Motifs

The relatively low number of bacterial Ser/Thr/Tyr phosphorylations makes it difficult to find statistically significant phosphorylation motifs. To our knowledge, only four phosphoproteomic works reported phosphorylation motifs in bacteria: the motifs XααααTX(X/V)ϕ(P/R)I (α is an acidic residue, and ϕ is a large hydrophobic residue) [3], and EXXpT, PpT and pTXp [38] were found in *Mycobacterium tuberculosis*; PFxFA[T/S]GY was described in *Sinorhizobium meliloti* [33]; and X(pT)xEx was identified in *Streptomyces coelicolor* [17]. It is clear that new workflows need to be explored to identify bacterial Ser/Thr/Tyr phosphorylation motifs. It may be interesting to combine all of the bacterial phosphorylated orthologue sequences, perhaps separated into different taxonomic groups, in the same motif search. In addition, the search algorithms might be modified to mine phosphorylation motifs in the reduced bacterial phosphoproteomes. Until significant bacterial Ser/Thr/Tyr phosphorylation motifs are found, it will be difficult to create robust bioinformatics tools to perform reliable in silico bacterial phosphorylation predictions.

### 3.3. Bacterial Processes Demonstrated to be Modulated by Ser/Thr/Tyr Phosphorylation

While Ser/Thr/Tyr phosphorylation is present in all of the analysed bacteria (Table 1, Table 2 and Table 3; Figure 1), there are very few bacterial processes that have been demonstrated to be regulated by Ser/Thr/Tyr phosphorylation. Some of the best characterised bacterial activities modulated by Ser/Thr/Tyr phosphorylation are cell wall metabolism, transcription and protein synthesis. Ser/Thr/Tyr kinases are required to modulate the activity of *Bacillus* cell wall hydrolases, in response to peptidoglycan fragments during spore germination [53] and the vegetative stage [54]. The Ser and Thr phosphorylation of *Deinococcus radiodurans* FtsA and FtsZ cell division proteins affects their functional interactions [55]. DivIVA, the key protein controlling apical growth in the mycelial bacterium *Streptomyces*, is modulated by the Ser/Thr kinase AfsK [56]. The *Streptococcus suis* DivIVA orthologue was also demonstrated to be modulated by a Ser/Thr kinase [57].

Other important bacterial processes were demonstrated to be regulated by Ser/Thr/Tyr phosphorylation. Quorum sensing was described to be regulated by phosphorylation in the marine pathogen, *Vibrio alginolyticus* [58,59]. *Streptococcus suis* growth is modulated by phosphorylation [60]. Gene expression was reported to be modulated by Ser/Thr/Tyr phosphorylation in *Staphylococcus aureus* [61] and *Streptococcus* [62]. The Tu elongation factor is modulated by Thr phosphorylation in *Mycobacterium tuberculosis* [63]. Photosynthesis was demonstrated to be modulated by Ser/Thr/Tyr phosphorylation in the model cyanobacterium, *Synechocystis* sp. [64]. The phosphorylation of the β subunits of phycocyanins affects the energy transfer and the state transition of *Synechocystis* photosynthesis [64]. Bacterial virulence can also be modulated by phosphorylation. Phosphorylation of the AmpC β-lactamase reduces β-lactamase activity and increases antibiotic resistance in *Acinetobacter baumannii* [6]. *Xanthomonas citri* virulence is activated by the phosphorylation of the Lon protease, which stabilises HrpG, the master regulator of type III secretion systems in this pathogenic bacterium [65].

## 4. Bacterial Histidine Protein Phosphorylation

Histidine phosphorylation was first demonstrated in bacterial two-component systems in 1980 [66]. Since then, several descriptions of two-component system signalling in prokaryotes have been reported. Histidine kinases are the most abundant protein kinases in bacteria. For instance, *Streptomyces coelicolor*, a bacterium harbouring the largest amount of eukaryotic-type Ser/Thr/Tyr kinases [17], has 47 Ser/Thr/Tyr kinases and 149 histidine kinases. By contrast, to the best of our knowledge, only a single His kinase in eukaryotic cells, which is highly conserved in eukaryotes and implicated in suppressing tumour metastasis, has been characterised [4,67].

### 4.1. Methodological Challenges

The histidine phosphate linkage has a half-life of about 30 min at pH 3 [68], which makes histidine phosphorylation incompatible with most LC-MS/MS analyses. Consequently, the characterisation of histidine phosphoproteomes remains a difficult challenge. To the best of our knowledge, there are only three reports describing His phosphoproteomic analyses on bacteria by means of standard shotgun phosphoproteomics, i.e., using acidic solvents [5,6,7]. Lai et al. [5] analysed the histidine phosphoproteome of nine bacteria, identifying seven and 31 phosphopeptides per bacterium [5]. They identified some pathogenicity proteins that were phosphorylated at histidine in *Acinetobacter baumannii*, *Klebsiella pneumoniae*, *Vibrio vulnificus* and *Helicobacter pylori* [5]. Lin et al. [7] and Lai et al. [6] analysed histidine phosphorylation together with the phosphorylation of Ser, Thr, Tyr and Asp in *Klepsiella pneumoniae* and *Acinetobacter baumanii*, respectively. They found that 12.9% (in *Klebsiella*) and 4.9% (in *Acinetobacter*) of the identified phosphorylations correspond to pHis. Given that acid buffers were used in these three works [5,6,7], there are probably many phosphohistidines that were not identified. These works give rise to the interesting question of whether the raw data of the other Ser/Thr/Tyr bacterial phosphoproteomic works can be processed, setting pHis as a post-translational modification, to identify novel histidine phosphorylations.

Histidine phosphorylation has constituted a methodological challenge for decades. The recent development of 1- and 3-pHis monoclonal antibodies [69,70] has contributed, at least in part, to overcoming this important methodological drawback. Kleinnijenhuis et al. [71] proposed to develop a fast LC method or nonacidic solvent systems to protect phosphohistidines from acidic dephosphorylation. Reverse-phase chromatography at a neutral or basic pH value, combined with the use of negative-ion-mode MS, might also be an alternative.

### 4.2. Bacterial Pathways Modulated by Histidine Phosphorylation

Bacterial two-component systems are the most important form of bacterial signal transduction. Canonical two-component systems are formed by a sensor histidine kinase, usually a transmembrane receptor, and a response regulator, usually a transcriptional regulator. The sensor histidine kinase transfers the phosphoryl group to the response regulator modulating its activity. Bacterial genomes harbour huge amounts of two-component signalling systems. For instance, *Streptomyces coelicolor* harbours more than 100 two-component signalling systems (www.sanger.ac.uk), many of which regulate secondary metabolism and antibiotic production [72]. Bacterial two-component systems modulate important cellular processes, such as photoreception [73], quorum sensing [74], temperature sensing [75] and plant-bacteria interactions [76].

Histidine kinases belonging to two-component systems can be predicted in silico, since the kinases and their response regulator genes are usually located adjacently in a genome. Once identified, their putative response regulators and functions can be studied. However, there are bacterial histidine kinases beyond two-component systems, of which the biological function is much more difficult to characterise. These latter types of kinases, which are not associated with the known response regulators, also show important regulatory activities, such as chemotaxis [77] or nucleoside metabolism [75].

## 5. Conclusions

The huge advances in LC-MS/MS methodologies and phosphopeptide enrichment, developed over the last 20 years, has made the study of large datasets of Ser/Thr/Tyr phosphopeptides possible, mainly in eukaryotes, but also in bacteria. Ser/Thr/Tyr protein phosphorylation in bacteria is dramatically lower than that in eukaryotes. However, this important post-translational modification is present in all the analysed bacteria (Table 1, Table 2, Table 3 and Table 4) and affects important cellular processes. While Ser/Thr/Tyr phosphorylation exists and is important in bacteria, there is a consensus that histidine phosphorylation is the most abundant protein phosphorylation in bacteria. However, histidine phosphoproteomes remain elusive due to the reduced phosphohistidine half-life under the acidic pH levels used in the shotgun phosphoproteomic procedures. Considering the fast and continuous advance in LC-MS/MS-based phosphoproteomic methodologies, it is expected that further innovations, such as the recent EasPhos platform developed by Humphrey et al. [78] and the development of workflows compatible with histidine phosphorylation stability, will allow for a better coverage of bacterial Ser/Thr/Tyr and His phosphoproteomes. Applying these kinds of methodologies to analyse bacterial phosphoproteomes might revolutionise our understanding of bacterial physiology.

## Figures and Tables

**Table 1:** Bacterial Ser/Thr/Tyr phosphoprotemic studies. Abbreviations: n.r., not reported; Ch, chemoheterotrophic.

Bacterium	Year	pSer (%)	pThr (%)	pTyr (%)	Reference
*Bacillus subtillis*	2007	69.2	20.5	10.3	[8]
*Escherichia coli* (*E. coli*)	2008	68	23	9	[9]
*Lactococcus lactis*	2008	46.5	50.6	2.7	[10]
*Klebsiella pneumoniae*	2009	31.2	15.1	25.8	[7]
*Pseudomonas aeruginosa/putida*	2009	52.8	36.1	11.1	[11]
*Halobacterium salinarum*	2009	84	16	0	[12]
*Mycobacterium tuberculosis*	2010	40	60	0	[3]
*Streptomyces coelicolor*	2010	34	52	14	[13]
*Streptococcus pneumoniae*	2010	47	44	9	[14]
*Bacillus subtilis*	2010	n.r.	n.r.	n.r.	[15]
*Neisseria meningitidis*	2011	n.r.	n.r.	n.r.	[16]
*Streptomyces coelicolor*	2011	46.8	48	5.2	[17]
*Listeria monocytogenes*	2011	93	43	7	[18]
*Helicobacter pylori*	2011	42.8	38.7	18.5	[19]
*Clostridium acetobutylicum*	2012	40	50	10	[20]
*Rhodopseudomonas palustris* (*Ch*)	2012	63.3	16.1	19.4	[21]
*Thermus thermophilus*	2012	65.3	26	8.7	[22]
*Thermus thermophilus*	2013	57	36	7	[23]
*Synechococcus* sp.	2013	43.9	42.44	13.66	[24]
*E. coli*	2013	75.9	16.7	7.4	[25]
*Staphylococcus aureus*	2014	n.r.	n.r.	n.r.	[26]
*Acinetobacter baumanii* Abh12O-A2	2014	71.8	25.2	3.8	[27]
*Acinetobacter baumanii* ATCC 17879	2014	68.9	24.1	5.2	[27]
*Pseudomonas aeruginosa*	2014	49	24	27	[28]
*Listeria monocytogenes*	2014	64	31	5	[29]
*Saccharopolyspora erythraea*	2014	47	45	8	[30]
*Bacillus subtilis*	2014	74.6	18.6	7.3	[31]
*Chlamydia caviae*	2015	n.r.	n.r.	n.r.	[32]
*Sinorhizobium meliloti*	2015	63	28	5	[33]
*E. coli*	2015	n.r.	n.r.	n.r.	[34]
*Bacillus subtilis*	2015	n.r.	n.r.	22.6	[35]
*Synechocystis* sp.	2015	n.r.	n.r.	n.r.	[36]
*Acinetobacter baumannii* SK17-S	2016	47	27.6	12.4	[6]
*Acinetobacter baumannii* SK17-R	2016	41.4	29.5	17.5	[6]
*Mycobacterium smegmatis*	2017	27.79	73.97	1.24	[37]
*Mycobacterium tuberculosis*	2017	68	29	3	[38]
*Microcystis aeruginosa*	2018	n.r	n.r.	n.r.	[39]
*Streptomyces coelicolor*	2018	50.6	47.4	2	[40]
*Zymomonas mobilis*	2019	73	21	6	[41]
*Streptococcus thermophilus*	2019	43	33	23	[42]
**Average**		**55.9**	**34.1**	**9.9**	

**Table 2:** 2DE gel-based bacterial phosphoproteome studies.

Bacterium	Year	Phosphoproteins	Phosphorylation Sites	Phosphoproteome	Reference
*Neisseria meningitidis*	2011	51	n.r.	Many biological processes	[16]
*Staphylococcus aureus*	2014	103	76	Pathogenicity and virulence	[26]
*Chlamydia caviae* (elementary body)	2015	42	n.r.	Virulence	[32]
*Chlamydia caviae* (reticulate body)	2015	34	n.r.	Virulence	[32]

**Table 3:** LC-MS/MS-based nonquantitative bacterial phosphoproteome studies. Abbreviation: Ph, photoheterotrophic.

Bacterium	Year	Phosphoproteins	Phosphorylation Sites	Phosphoproteome	Reference
*Bacillus subtilis*	2007	78	78	Carbohydrate metabolism	[8]
*E. coli*	2008	79	81	Similar to *Bacillus*	[9]
*Lactococcus lactis*	2008	63	79	Over-representation of phosphothreonines	[10]
*Klebsiella pneumoniae*	2009	81	93	Capsular biosynthesis	[7]
*Pseudomonas aeruginosa*	2009	39	61	Motility, transport and pathogenicity	[11]
*Pseudomonas putida*	2009	59	55	Several biochemical pathways	[11]
*Halobacterium salinarum*	2009	26	31	Phosphoproteome in Archaea	[12]
*Mycobacterium tuberculosis*	2010	301	500	Several biochemical pathways	[3]
*Streptomyces coelicolor*	2010	40	46	Housekeeping proteins	[13]
*Streptococcus pneumoniae*	2010	84	163	Carbon/protein/nucleotide metabolisms, cell cycle and division	[14]
*Listeria monocytogenes*	2011	112	143	Virulence, translation, carbohydrate metabolism and stress response	[18]
*Helicobacter pylori*	2011	67	126	Virulence	[19]
*Clostridium acetobutylicum*	2012	61	107	Carbon metabolism	[20]
*Rhodopseudomonas palustris* (*Ch*)	2012	54	63	Carbon metabolism	[21]
*Rhodopseudomonas palustris* (*Ph*)	2012	42	59	Carbon metabolism	[21]
*Thermus thermophilus*	2012	48	46	Wide variety of cellular processes	[22]
*Thermus thermophilus*	2013	53	67	Central metabolic pathways and protein/cell envelope biosynthesis	[23]
*Synechococcus* sp.	2013	245	410	Two-component signalling pathway and photosynthesis	[24]
*Acinetobacter baumanii* Abh12O-A2	2014	70	80	Pathogenicity and drug resistance	[27]
*Acinetobacter baumanii* ATCC 17879	2014	41	48	Several biochemical pathways	[27]
*Pseudomonas aeruginosa*	2014	28	59	Extracellular virulence factors	[28]
*Sinorhizobium meliloti*	2015	77	96	Rhizobial adaptation	[33]
*Microcystis aeruginosa* (nontoxic)	2018	37	n.r.	Several biochemical pathways	[39]
*Microcystis aeruginosa* (toxic)	2018	18	n.r.	Regulation of toxin generation	[39]

**Table 4:** LC-MS/MS-based quantitative bacterial phosphoproteome studies.

Bacterium	Year	Phosphoproteins	Phosphorylation Sites	Phosphoproteome	Method	Reference
*Bacillus subtilis*	2010	27	45	Phosphoproteome changes in different media	SILAC	[15]
*Streptomyces coelicolor*	2011	127	289	Sporulation factors, transcriptional regulators, protein kinases and other regulatory proteins	Label-free	[17]
*E. coli*	2013	133	108	Stationary phase	SILAC	[25]
*Bacillus subtilis*	2014	141	177	Stationary phase	SILAC	[31]
*Listeria monocytogenes*	2014	191	242	Purine biosynthesis regulated by PrfA phosphorylation	SILAC	[29]
*Saccharopolyspora erythraea*	2014	88	109	Carbon metabolism, environmental stress and protein synthesis affected by phosphorylation	SRM	[30]
*E. coli*	2015	71	n.r.	Phosphorylation varied during development	SRM	[34]
*Bacillus subtilis*	2015	124	155	Spore-specific determinants	Label-free	[35]
*Synechocystis* sp.	2015	188	262	Increased phosphorylation during nitrogen limitation	Dimethyl	[36]
*Acinetobacter baumannii* SK17-S	2016	248	410	Antibiotic resistance	Label-free	[6]
*Acinetobacter baumannii* SK17-R	2016	211	285	Antibiotic resistance	Label-free	[6]
*Mycobacterium smegmatis*	2017	154	224	Transmembrane proteins	Label-free	[37]
*Mycobacterium tuberculosis*	2017	257	512	Virulence	Tandem mass tag (TMT)	[38]
*Streptomyces coelicolor*	2018	48	85	Regulatory proteins	TMT	[40]
*Zymomonas mobilis*	2019	125	177	N2 fixing regulated by phosphorylation	Label-free	[41]
*Streptococcus thermophilus*	2019	106	161	Divisome proteins phosphorylated by the PknB kinase	Dimethyl	[42]

## Abbreviations

LC-MS/MS	liquid chromatography tandem mass spectrometry
IMAC	immobilised metal affinity chromatography
CPP	calcium phosphate precipitation
SILAC	stable isotope labelling by amino acids in cell culture
TMT	tandem mass tag
sMRM	scheduled multiple reaction monitoring
