# Correction: Yagüe, P., et al. Goals and Challenges in Bacterial Phosphoproteomics. *Int. J. Mol. Sci.* 2019, *20*, 5678

**DOI:** 10.3390/ijms21249381

**Published:** 2020-12-09

**Authors:** Paula Yagüe, Nathaly Gonzalez-Quiñonez, Gemma Fernández-García, Sergio Alonso-Fernández, Angel Manteca

**Affiliations:** Área de Microbiología, Departamento de Biología Funcional, IUOPA, ISPA, Facultad de Medicina, Universidad de Oviedo, 33006 Oviedo, Spain; paula.yague@gmail.com (P.Y.); natygq@gmail.com (N.G.-Q.); gemmafg06@hotmail.com (G.F.-G.); sergioalonsofernandez@gmail.com (S.A.-F.)

The authors wish to make the following corrections to this paper [1]:

1. The author name “Gemma Fernánez-García” should be “Gemma Fernández-García”.
2. Page 2, first line: “257 proteins” should be “301 proteins”.

The authors would like to apologize for any inconvenience caused to the readers by these changes.
